# Tracking Personal Health-Environment Interaction with Novel Mobile Sensing Devices

**DOI:** 10.3390/s18082670

**Published:** 2018-08-14

**Authors:** Yue Deng, Nai-Yuan Liu, Francis Tsow, Xiaojun Xian, Rosa Krajmalnik-Brown, Nongjian Tao, Erica Forzani

**Affiliations:** 1 School for Engineering of Matter, Transport and Energy, Arizona State University, Tempe, AZ 85287, USA; ydeng29@asu.edu (Y.D.); nliu26@asu.edu (N.-Y.L.); 2 Center for Bioelectronics and Biosensors, Biodesign Institute, Arizona State University, Tempe, AZ 85287, USA; frant@earthlink.net (F.T.); xiaojun.xian@asu.edu (X.X.); 3 Swette Center for Environmental Biotechnology, Biodesign Institute, Arizona State University, Tempe, AZ 85287, USA; Dr.Rosy@asu.edu; 4 School of Sustainable Engineering and the Built Environment, Arizona State University, Tempe, AZ 85287, USA

**Keywords:** volatile organic compounds (VOCs), resting metabolic rate (RMR), mobile sensors, environmental exposure

## Abstract

The development of connected health devices has allowed for a more accurate assessment of a person’s state under free-living conditions. In this work, we use two mobile sensing devices and investigate the correlation between individual’s resting metabolic rate (RMR) and volatile organic compounds (VOCs) exposure levels. A total of 17 healthy, young, and sedentary office workers were recruited, measured for RMR with a mobile indirect calorimetry (IC) device, and compared with their corresponding predicted RMR values from the Academy of Nutrition and Dietetics’ recommended epidemiological equation, the Mifflin–St Jeor equation (MSJE). Individual differences in the RMR values from the IC device and the epidemiological equation were found, and the subjects’ RMRs were classified as normal, high, or low based on a cut-off of ±200 kcal/day difference with respect to the predicted value. To study the cause of the difference, VOCs exposure levels of each participant’s daytime working environment and nighttime resting environment were assessed using a second mobile sensing device for VOCs exposure detection. The results showed that all sedentary office workers had a low VOCs exposure level (<2 ppmC), and there was no obvious correlation between VOCs exposure and the RMR difference. However, an additional participant who was a worker in an auto repair shop, showed high VOCs exposure with respect to the sedentary office worker population and a significant difference between measured and predicted RMR, with a low RMR of 500 kcal/day difference. The mobile sensing devices have been demonstrated to be suitable for the assessment of direct information of human health–environment interactions at free-living conditions.

## 1. Introduction

Connected health is an emerging field that has been reported as a social-technical model for healthcare management and delivery [1]. It is also extended to include the effect of environmental factors on specific health conditions. Connecting the effect of environmental pollutants to personal health opens the possibility of addressing important open questions in the research field of environmental health. To be able to monitor and track environment-health connections, technological challenges need to be overcome. First, robust real-time mobile sensing devices must be developed for the simultaneous monitoring of health and environmental parameters. Second, the mobile sensing devices must be accurate to ensure the validity of the collected data. Third, the mobile devices must have user-friendly interfaces so that users with relatively low levels of technical backgrounds can operate them effectively. Fourth, the data collected from the devices must be reliable to assure sensing information is captured during the periods of use. Finally, the data analysis needs to provide meaningful output information [2]. In this work, two mobile sensing devices built for the simultaneous capture of health and environmental parameters were utilized and demonstrated how sensor data can be used to explore new correlations. The correlation between personal resting metabolic rate (RMR) and the corresponding exposure to volatile organic compounds (VOCs) is investigated and discussed.

Resting metabolic rate (RMR) is a parameter that indicates a person’s daily energy expenditure (kcal/day or kJ/day) [3], and it is used to properly estimate individual caloric needs [4,5] for effective body weight management [6]. Although epidemiological equations have been developed for calculating RMR [7], it is necessary for an individual to assess their RMR value by direct measurements [8], because one’s RMR value depends on many factors such as body composition, diet, hormone levels, environment exposure, and so forth [3]. Several methods used to measure an individual’s RMR have been developed in the past decades, including direct calorimetry [9] and indirect calorimetry (IC) [10]. Laboratory bench-based instruments to measure RMR have been developed, such as the metabolic cart [3]. However, these bulky instruments are developed for research and professional use and are far too large to accurately measure RMR in free-living conditions. As reported by our group and others, although in the population, on average, there is no statistically significant difference between measured RMR and predicted RMR, *significant and clinically relevant differences are found at the individual level* [11,12]. In recent years, handheld and mobile devices have been developed for personal use [13,14], which make it practical for individuals to monitor their RMR daily and design evidence-based personal health plans. 

RMR has been determined to be affected by exposure to environmental persistent organic pollutants (POPs). These chemicals are resistant to degradation and can adversely affect human health and the environment [15], degrading the activity of the oxidative enzymes responsible for energy production [16,17,18,19,20,21]. Furthermore, exposure to POPs has been linked to weight gain [17]. 

**Figure 1:**
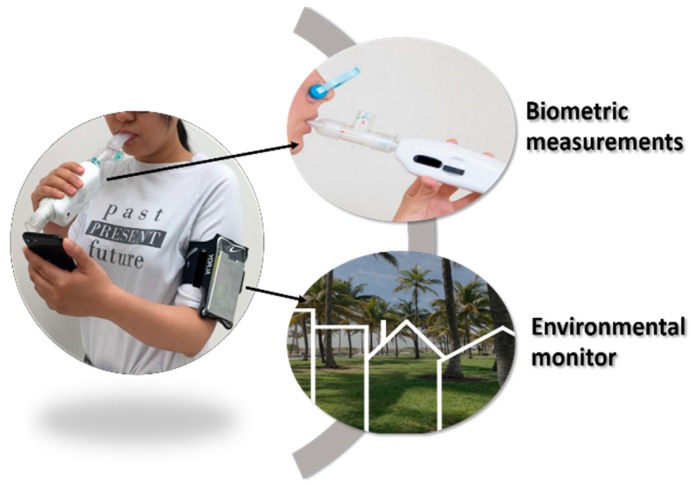
A participant using the mobile indirect calorimeter for assessment of resting metabolic rate (RMR, biometric measurement) simultaneously with a mobile environmental volatile organic compounds (VOCs) monitor worn in the arm band.

In order to explore potential connections between RMR and environmental pollutant exposure, in this paper, we focus on the assessment of volatile organic compounds (VOCs)—specifically detected by our developed VOCs personal monitor—which are long-chain alkanes, alkenes, and aromatic compounds (see below more details). VOCs are among the top air pollutants in both indoor and outdoor environments [22] that present harm to human health and the environment [23]. The exposure limit to a variety of VOCs (i.e., benzene, toluene, trichloroethylene, and tetrachloroethylene) has been listed by the World Health Organization guidelines for indoor air quality [24]. For different chemicals, the recommended exposure limit ranges from 2.3 μg/m^3^ to 0.25 mg/m^3^; however, for carcinogens such as Benzene, no safe exposure limit could be concluded. Furthermore, VOCs in blood can diffuse across the pulmonary alveolar membrane. Given the chemical similarity of VOCs (e.g., long-chain alkanes, alkenes, and aromatic compounds) with POPs, we hypothesize that correlations of RMR level and exposure level to VOCs can be easily assessed by using novel mobile sensing devices [25]. To the best of our knowledge, we are unaware of any report that directly associates VOCs exposure with RMR. Therefore, we believe it is important to demonstrate the feasibility of the assessment of data with mobile sensing devices *to explore the hypothesis of whether the environmental VOCs exposure at a personal level has any effect on an individual’s RMR and whether it, therefore, influences the capability of daily energy burning*. In this work, *the goal is to demonstrate the simultaneous use of two mobile sensing devices, so-called trackers, to directly monitor personal VOCs exposure and an individual’s RMR*. Figure 1 shows a picture of the mobile sensing devices used to assess RMR and VOCs exposure levels, simultaneously. We tracked 17 sedentary office workers’ VOCs exposure with a portable VOC monitor. During the same period, their RMRs were measured using the mobile indirect calorimeter. Furthermore, we tracked an additional participant, who was a worker in a mechanic shop. The results show that real-time mobile sensing devices designed for connected health could achieve unprecedented data assessment. 

## 2. Materials and Methods

### 2.1. Participants

A total of 36 participants were recruited from the greater Phoenix area, AZ, USA in 2016. The recruitment criteria included overall good health, free from any medications that might affect metabolic rate, and no tobacco use. Female subjects who were pregnant and/or lactating were excluded. We obtained the Arizona State University Institutional Review Board approval prior to the study (IRB protocol # 1012005855 for the RMR measurement and IRB protocol # 1304009100 for the VOCs measurement). All the participants provided written informed consent before participating in the study for both IRB protocols.

### 2.2. Measurements

#### 2.2.1. Anthropometric Measurements

Physical parameters, including height, weight, gender, age, and body fat percentage (BF %) were recorded. Body compositions were determined by a Tanita bio-impedance scale (model: BC-554 IRONMAN^®^ Body Composition Monitor, Tanita, Tokyo, Japan). Height was measured with a wall-mounted stadiometer. These parameters were used to calculate RMR from the epidemiological equation. A summary of all the sedentary office workers recruited for the study is shown in Table 1. As mentioned in the introduction, all the participants were sedentary office workers, except one participant who was an auto mechanic worker. This specific participant is a 23-year old male. His body mass index (BMI) was 30.6 kg/m^2^ with a weight of 93.6 kg and a height of 1.75 m.

#### 2.2.2. Resting Metabolic Rate Measurements

RMR was measured using a portable indirect calorimeter Breezing^®^ from Tempe, AZ, USA. The Breezing^®^ device evaluates energy expenditure (EE) by measuring the rate of oxygen consumption and carbon dioxide generation in breath [26]. Its detection mechanism relies on a flow meter for the determination of breath flow rate and a chemical sensing cartridge for the measurement of oxygen and carbon dioxide in breath. As reported previously [13,27], the device connects wirelessly to a mobile device via Bluetooth^®^. The validation of this device has been previously described [13]. A quick response (QR) code with pre-calibrated sensor information was scanned with a cell phone camera, and the calibration factors for the single-use sensor cartridge were applied to the measurement.

The participants were required to adhere to the following pre-test conditions: no food or caffeine intake in the past 4 h, no strenuous exercise performed for the past 12 h, and no moderate exercise performed 4 h before the test. The participants were introduced to the testing procedure in the beginning. Physical parameters were then recorded. Three RMR measurements were done in the same morning once the resting state was assured. All the participants adhered to the testing instructions.

During the measurement, the participants breathed through a disposable mouthpiece attached to the Breezing^®^ device. The data received on the mobile device was processed and displayed on the user interface. The Weir equation was used to determine RMR from the measured oxygen consumption and CO_2_ production rates [26].

#### 2.2.3. VOCs Exposure Measurements

A mobile low-cost VOCs sensing device for free-living conditions has been reported before by our group [28,29,30,31]. The sensing device works with a frequency-based sensing mechanism, which utilizes a mass sensitive piezoelectric resonator with a selective polymer (molecularly imprinted polymer) modified quartz tuning fork. The change in the resonant frequency of the sensor can be related to with the total VOCs concentration. Each measurement takes approximately 3 min. During the first 2 min, a purging period takes place. Ambient air passes through a zeroing filter generating clean air and allowing the sensor to generate a stable baseline. In the third minute, a sampling period takes place. Ambient air is directly sampled into a channel where only particles in the incoming air are filtered. VOCs in the air are detected via a resonant frequency change assessed with respect to the baseline established in the purging period. The mobile VOCs sensing device connects wirelessly to an Android smart phone via Bluetooth^®^. The validation tests of the VOC monitor were reported in our previous publications [28,29,30,31]. A total of 18 participants out of the 36 participants were randomly selected to have their VOCs exposure measured throughout the day. The participants wore the portable monitor in an armband and had their VOCs exposure assessed for two one-hour-long periods during the day and at night, respectively. The participants spent their daytime in cubic offices and open space offices, which have a positive pressure and high air exchange rates.

#### 2.2.4. Epidemiological Equation

The Academy of Nutrition and Dietetics’ recommended equation for estimating RMR, the Mifflin–St Jeor equation (MSJE), was used to predict the participants’ RMR from their age, gender, weight, and height. The equation is a result of the regression from 498 healthy individuals performed on indirect calorimetry measurements with metabolic carts [7]: RMR (kcal/day) = 9.99 × weight (kg) + 6.25 × height (cm) − 4.92 × age (*y*) + 166 × sex (males, 1; females, 0) – 161.

In this study, the MSJE has been used as an epidemiological equation representative of the population average RMR. Therefore, it has been used for RMR calculation and compared with the measured RMR values.

## 3. Results

### 3.1. RMR Measurement Results Using Mobile IC and Comparison with Calculated with MSJE

**Figure 2:**
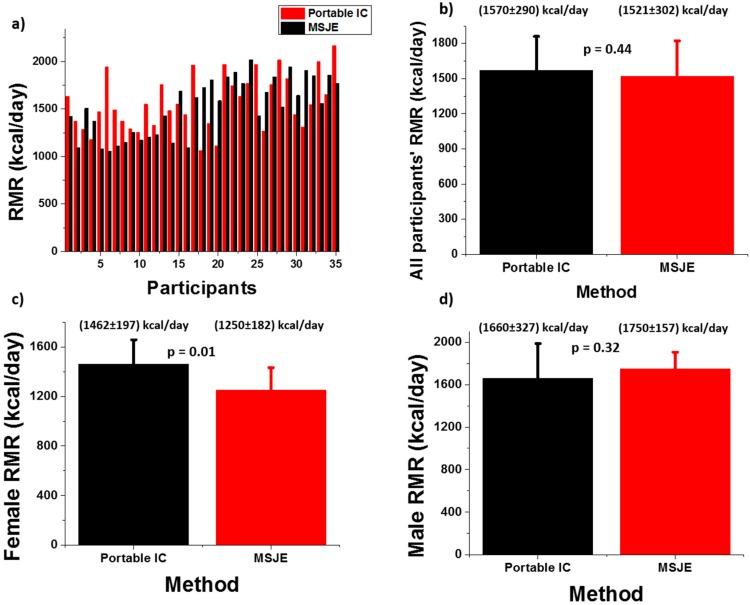
RMR results comparison between mobile indirect calorimeter (IC) measurement and the Mifflin–St Jeor equation (MSJE) prediction [12]. (**a**) Raw RMR data from the two methods; (**b**) Averaged RMR comparison between the two methods for all the participants; (**c**) Averaged RMR comparison between the two methods for female participants only; and (**d**) Averaged RMR comparison between the two methods for male participants only.

The RMR of every participant was measured three times using the mobile IC, and the results were averaged. The RMR value was calculated using the MSJE from the collected physical parameters, and a comparison of the measured and calculated RMR was performed. Figure 2 shows a summary of the results.

The raw RMR values from the two methods are presented in Figure 2a, and the averaged RMRs for different participant groups (all participants, female and male) are shown in Figure 2b–d. Paired *t*-tests were performed, and an α = 0.01 and *p* <0.01 were set for statistical significance. The *p* values for the three paired *t*-tests were 0.44, 0.01, and 0.32 respectively. The results showed that there is no significant difference between the predicted and calculated RMRs for any of the groups. 

### 3.2. RMR Difference between the Two Methods

**Figure 3:**
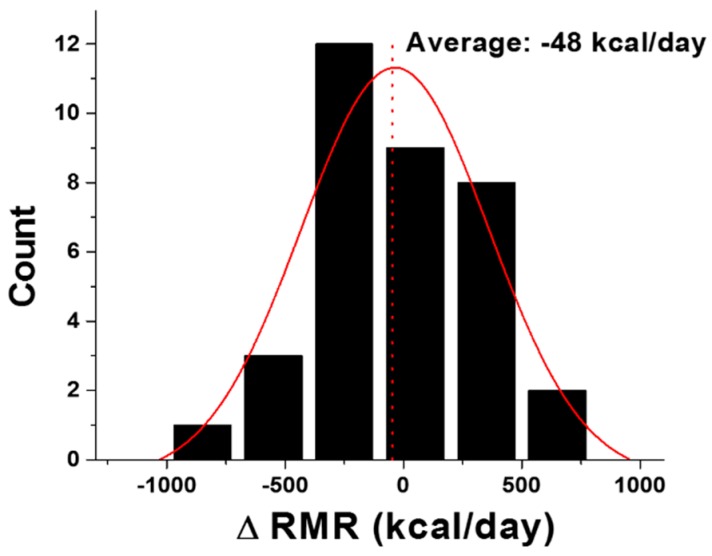
Distribution of RMR difference between the two methods, ∆RMR = RMR from the MSJE − RMR from the mobile IC.

Based on this study and our previously published results [12], we have concluded that there is no statistical difference in the RMR values from the portable IC measurement and the MSJE calculation. Because the MSJE was derived from results assessed with metabolic carts, the finding indicates that the mobile IC device is accurate. However, as shown in Figure 3, the distribution of differences between these two methods (∆RMR = RMR from the MSJE − RMR from the portable IC) varies from individual to individual and ranged from −887 kcal/day to 665 kcal/day, which is a wide range with physiological significance. Taking into account the precision of the mobile IC and the potential intrinsic clinical fluctuations of the RMR measurements, a range of ±200 kcal/day for the difference was set to determine the agreement of the measured and calculated RMRs. Although the average difference is −48 kcal/day, which is quite close to equality (*x* = 0), the RMR differences for only 10 out of 35 participants (28.6%) fell within ±200 kcal/day. Because the MSJE is the result of a regression model of a large population study, it represents the average RMR value of the population and *is not expected to reflect an individual’s unique RMR*. Therefore, we can conclude that for a large portion of the studied group, the individual RMR differed from the MSJE predicted average by more than ±200 kcal/day.

### 3.3. VOCs Exposure Measurement

Based on the ∆RMR, defined as the difference between the RMR from MSJE and the RMR from the mobile IC, the participants were divided into three groups:

- Group A: “low RMR” group with measured RMR values smaller than the corresponding calculated RMR values by a difference of 200 or greater kcal/day;
- Group B: “normal RMR” group with measured RMR values equal to ±200 kcal/day of the corresponding calculated RMR values; and
- Group C: “high RMR” group with measured RMR values higher than the corresponding calculated RMR values by a difference of 200 or greater kcal/day.

As a result, 10 participants were found to be in Group A (low RMR group); 10 participants were found to be in Group B (normal RMR group); and 15 participants were found to be in Group C (high RMR). Among these participants, a total of 17 were recruited for VOCs exposure testing as follows: six participants were from Group A, six participants were from Group B, and five participants were from Group C. The number of participants was chosen based on power calculation analysis [32]. Assuming a typical mean exposure for VOCs of 1 ppmC, with a standard deviation of 0.5–0.6 ppmC (detection limit of the method), a sample size of five to six subjects would allow for discrimination of a mean exposure increase to a mean value of 2 ppmC (twice higher) with a power of 0.80 and an alpha of 0.05 [32]. The demography of these 17 participants is summarized in Table 2.

The participants were asked to wear the mobile VOCs monitor for about 1 h at work and 1 h at home for a total of 2 h in a day. The monitor was placed inside an armband, and real-time data was recorded on an Android phone. No discomfort or hindrance during their activity was reported. 

**Figure 4:**
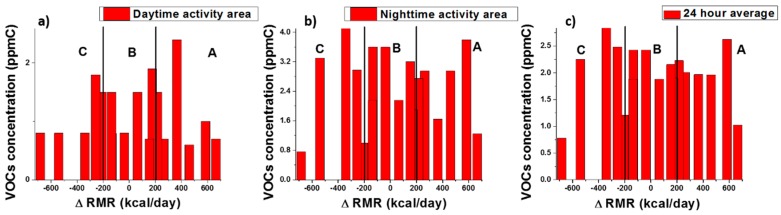
Average VOCs concentration for each test: (**a**) participants’ work area; (**b**) participants’ home; (**c**) prediction of 24-h exposure level by applying the average time spent in each location. A, B, and C indicate the group of participants with low, normal, and high RMR, respectively.

Figure 4 shows the summary of the VOCs exposure for all the subjects. The averaged total VOCs concentration (evaluated as ppmC) of each test was plotted against the ∆RMR of each participant. The daytime and nighttime VOCs concentrations are presented in Figure 4a,b. On average, the participants reported spending 10 h at work and over 12 h at home. To simplify the calculation for an individual’s 24-h hydrocarbon exposure, we used their average time spent at work and home as weights for the measured VOCs exposure level as shown in the equation below.

(1) $$\begin{array}{c} 24\;h\;exposure\;concentration\\ \;\;\;\;\;\;\;\;=\frac{10}{24}\times daytime\;area\;VOCs\;concentration\\ \;\;\;\;\;\;\;\;+\frac{14}{24}\times nighttime\;area\;VOCs\;concentration \end{array}$$

As reported in the previous publication, the total VOCs concentration obtained from this VOCs device is in terms of volume in total carbon concentration (ppmC) [33]. As seen in Figure 4, for most cases, the participants’ homes have higher VOCs concentrations compared with their work area. This may due to the better ventilation in most office buildings than in residential constructions [34,35]. 

## 4. Discussion

We applied ANOVA analysis [36] to quantitatively analyze the VOCs exposure concentration within the three groups. Table 3 shows the average VOCs exposure in each test and the statistics from the ANOVA analysis. The average VOCs exposures for all three groups under each condition were similar. The *p* values for all three conditions were higher than 0.01, indicating no significant difference within the three groups. In addition, the F values from the ANOVA analysis were smaller than the F critical values. Thus, the null hypothesis that there is no significant difference within the three groups is not rejected.

For the healthy, young, and sedentary office lifestyle population, there is no evidence in our study that suggests a correlation between the amount of VOCs exposure and the RMR difference between the measured RMR value and the predicted value from the equation. This is can be attributed to the fact that the VOCs exposures of these participants were not high, and the causes making the individuals have high, normal, or low RMR values were other than the exposure to VOCs.

**Figure 5:**
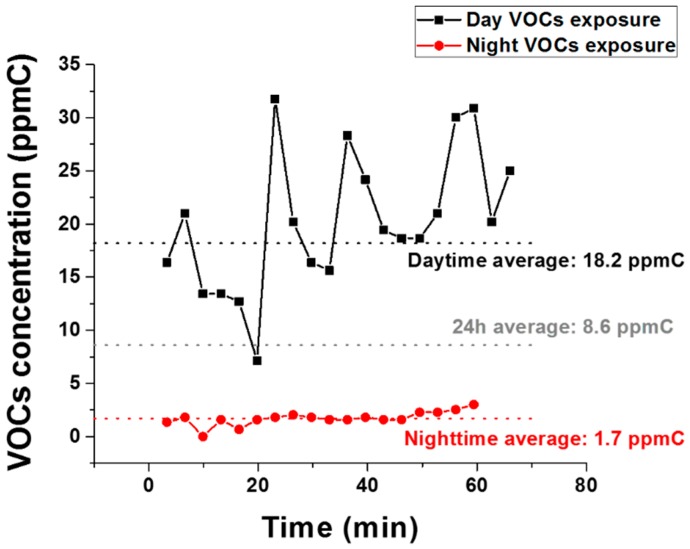
High VOCs exposure case. VOCs exposure concentration for the auto mechanic.

However, it is important to determine if the same result could be concluded for people who are consistently exposed to high VOC levels. To gain insights into that question, we also recruited one participant who works in an automobile repair shop. For this specific participant, the RMR value estimated using the MSJE was 2081 kcal/day, whereas the participant’s actual measured RMR using the portable IC was 1425 kcal/day; a difference greater than 500 kcal/day. As such, a fitness and dietary plan developed using the epidemiological equation (e.g., MSJE) would lead to a daily energy surplus and result in weight gain. Figure 5 shows the exposure level of this individual with an average value of 18.2 ppmC during work, which was much higher than the participant’s home averaged VOCs exposure concentration. This concentration was also much higher than all the other sedentary participants’ work exposure, while his home VOCs exposure concentration (1.7 ppmC) was comparable to the other 17 participants. Applying the same 24-h VOCs exposure equation to this dataset, this participant was exposed to 8.6 ppmC VOCs per day, which is three times higher than the sedentary office lifestyle participants.

From the single participant, we cannot conclude whether high level of VOCs exposure will have a long-term effect on RMR. However, this example draws attention to the importance of tracking personal exposure in real-time and demonstrates our protocol in studying health–environment interactions.

There are many factors that contribute to metabolic rates such as body composition, diet, and so forth [3]. It is a complex biometric that maybe influenced by many aliasing factors. The purpose of this study was to investigate the correlation between environmental VOCs exposure and RMR in a direct way, specifically, to preliminary measure different populations’ VOCs exposure concentration and their RMRs. 

In this study, the use of two portable devices enhanced our experimental proceedings as they are easy to operate and provide tracking features. Our approach using these mobile devices makes collecting real-time data at low cost and in free-living conditions feasible. Compared with traditional bulky lab-based instruments, mobile devices also provide real-time measurements.

Although we do not observe a significant VOCs exposure difference for the three groups of high, normal, and low RMR, it is noticed that the participants with high RMRs (group C) have relatively lower BMIs and body fat percentages with respect to the normal and low RMR groups (groups B and C) (Table 2). This may indicate that for populations exposed to a low VOCs level, the body composition may be still a dominating factor in determining people’s RMR values. Furthermore, the results from a single participant exposed to a high VOCs environment are a guide for future work towards the assessment of RMR and VOCs exposure levels of individuals who are being occupationally exposed to a high VOCs level (e.g., repair shop, gym) or live in highly polluted areas worldwide [37,38].

## 5. Conclusions

A mobile IC sensing device was used to assess individual’s RMR values, which were compared with calculated RMR, using a widely accepted predictive equation. Based on the differences between measured RMR and calculated RMR, we established three groups with normal RMR (±200kcal/day), high RMR (the measured RMR 200+ kcal/day higher than the calculated RMR), and low RMR (the measured RMR 200+ absolute kcal/day lower than the calculated RMR). We explored whether the level of RMR of each group had a correlation with the exposure level to VOCs. ANOVA results support the conclusion that there is no obvious correlation between the level of a person’s RMR (high, low, or normal) and that person’s VOCs’ exposure level. Furthermore, all the participants from the sedentary worker group showed relatively low VOC exposure levels (<2 ppmC). For this reason, the observed deviations of the participant’s measured RMR value from the epidemiologic expected values must be attributed to other factors. Nevertheless, the observation of a participant’s exposure level in a car repair shop showed significantly higher exposure than the sedentary population, and coincidentally, the measured RMR in one of the workers was classified as “low” when compared with the expected RMRs for individuals in a population of the same height, weight, gender, and age. The results show the synergic effect of the information collected by mobile real-time sensing devices to study host health–environment interactions. Further investigation of individuals with suspected high occupational or geographical VOCs exposure, as well as the intensity of activity, which drives higher breathing rates and exposure, is worthy of being explored in the future.

## Figures and Tables

**Table 1:** Sedentary office workers’ physical parameters.

	N	Age	Weight (kg)	Height (m)	BMI (kg/m^2^)	Fat (%)
All participants	35	27.8 ± 4.5 (23–47)	63.2 ± 13.4 (45–86)	1.69 ± 0.1 (1.42–1.91)	21.9 ± 2.4 (17.2–26.7)	16.9 ± 5.7 (6.0–29.2)
Males	19	29.5 ± 5.3 (23–47)	72.3 ± 9.99 (52.8–86)	1.78 ± 0.05 (1.70–1.91)	22.7 ± 2.6 (18.1–26.7)	13.8 ± 4.1 (6–19.6)
Females	16	25.2 ± 2.2 (23–30)	52.4 ± 7.33 (45–70)	1.58 ± 0.09 (1.42–1.75)	20.9 ± 1.9 (17.2–22.9)	20.6 ± 5.2 (13.2–29.2)

**Table 2:** VOCs exposure measurement participants’ physical parameters.

	N	Age	Weight (kg)	Height (m)	BMI (kg/m^2^)	Fat (%)
Group A	6	29.7 ± 9.0 (23–47)	70.7 ± 10.1 (59–81)	1.73 ± 0.08 (1.60–1.78)	23.8 ± 5.0 (18.1–32.8)	21.4 ± 13.7 (6–44.4)
Group B	6	27.6 ± 4.9 (23–36)	70.1 ± 9.8 (59–80)	1.71 ± 0.09 (1.60–1.83)	23.7 ± 1.6 (22.5–26.6)	21.5 ± 5.0 (17.3–28.2)
Group C	5	28.4 ± 3.9 (25–34)	56.5 ± 7.4 (50–65)	1.67 ± 0.08 (1.60–1.75)	20.3 ± 1.1 (18.4–21.1)	15.7 ± 5.6 (9.5–18.0)

**Table 3:** Average VOCs exposure in sedentary office workers and ANOVA analysis.

	Daytime Activity Area VOCs Exposure (ppmC)	Night Activity Area VOCs Exposure (ppmC)	24-h Average VOCs Exposure (ppmC)
Group A—low RMR	1.15 ± 0.63	2.56 ± 0.48	1.97 ± 0.48
Group B—normal RMR	1.20 ± 0.45	2.77 ± 0.24	2.11 ± 0.24
Group C—high RMR	1.14 ± 0.43	3.05 ± 1.30	2.25 ± 1.30
*p* value	0.98	0.88	0.86
F	0.018	0.134	0.147
F critical	3.74	3.74	3.74
